# Enhanced triglyceride adsorption by steam-activated bamboo charcoal based on molecular dynamics investigations

**DOI:** 10.1038/s41598-024-56902-9

**Published:** 2024-03-14

**Authors:** Hegang Zhu, Sheng Zhang, Honghui Zheng, Guifeng Wang

**Affiliations:** Central South University of Forestry and Technology, Changsha, 410004 China

**Keywords:** Biomaterials, Structural materials

## Abstract

In this study, ordinary bamboo charcoal was activated at 750 °C with a steam flow rate of 6.25 L/min for 1.5 h. The effects of triglyceride adsorption by activated bamboo charcoal were investigated using an orthogonal design, and the adsorption mechanism was explored through molecular dynamics. Experimental results revealed that the adsorption capacity of activated bamboo charcoal for triglycerides reached 27.0%. The activated bamboo charcoal exhibited a specific surface area of 560.0 m^2^/g. The average pore diameter of activated bamboo charcoal was 1.6 nm, whereas that of ordinary bamboo charcoal was 7.2 nm. Molecular dynamics simulations revealed an interaction energy of − 145.12 kcal/mol between the molecular layers of activated bamboo charcoal and the triglyceride molecules, as well as an interaction energy of − 132.73 kcal/mol between the molecular layers of ordinary bamboo charcoal and the triglyceride molecules. The quantity of triglyceride molecules adsorbed by activated bamboo charcoal per gram was estimated to be 1.77 × 10^21^ while ordinary bamboo charcoal could adsorb merely 1.56 × 10^19^ triglyceride molecules per gram. This stark contrast in adsorption capacity underscores the superior performance of activated bamboo charcoal than its counterpart.

## Introduction

In humans sebum is an oily substance secreted by the skin^1^. Excessive sebum secretion can lead to enlarged pores, oily skin, and a dull complexion^2,3^. Moreover, an accumulation of sebum can promote microbial overgrowth and lipid peroxidation, resulting in skin disorders such as acne^1,4,5^. Hence, it is crucial to regulate the sebum content of the skin. Sebum consists of 57% triglycerides, 26% wax esters, 12% squalene, and 2% cholesterol^6^. Triglycerides represent a major component of sebum; reducing their levels can decrease overall sebum production.

Existing methods to control excessive sebum secretion primarily involve using specific chemical compounds to modulate the endocrine system, such as isotretinoin^7^ and spironolactone^8^. However, isotretinoin can cause skin and mucous membrane dryness^9^, while prolonged spironolactone use can lead to menstrual irregularities, frequent urination, dizziness, hedaches, nausea, and vomiting^10^. The method of physically adsorbing sebum is safer than that of the above, for it does not affect the endocrine system.

Bamboo charcoal is a good physical adsorption material. It is produced by high-temperature pyrolysis of bamboo, boasts a large surface area and well-developed porosity and is biocompatible^11^. However, its adsorption capacity is limited^12^. Bamboo charcoal can be further activated to enhance its adsorption ability^13^ through physical or chemical activation methods. Chemical activation requires the use of activating agents (KOH, H_3_PO_4_, ZnCl_2_)^14,15^, but residual agents pose safety risks to the skin. Physical activation involves steam activation^16^, which ensures safety by not introducing additional chemical components to the activated bamboo charcoal.

Molecular dynamics (MD) serves as an important tool for depicting dynamic processes in adsorption systems^17^. Molecular simulation techniques build microscopic system structures and model molecular dynamic behaviors, providing insights into material properties at the molecular level and unveiling experimental mechanisms at the microscopic level^17^.

Currently, there are no reports on activated bamboo charcoal's capacity to adsorb skin sebum. This study employs steam-activated bamboo charcoal to investigate its effectiveness in adsorbing triglycerides. Utilizing Molecular Dynamics, the study examines the dynamic process of triglyceride adsorption on activated bamboo charcoal from a microscopic perspective, elucidating the mechanism behind this adsorption process.

## Materials and methods

### Preparation of steam-activated bamboo charcoal

Normal bamboo charcoal (procured from Hunan Xinsheng Bamboo Industry Co., Ltd., Yiyang City, China) was subjected to activation in a BJXG-8–10 atmosphere rotary furnace. Steam was employed as the activating agent at a temperature of 750 °C, with a controlled steam flow rate of 6.25 L/min. Activation was conducted for 1.5 h, resulting in the production of steam-activated bamboo charcoal^18^.

### Orthogonal experimental design for triglyceride adsorption by activated bamboo charcoal

An orthogonal experimental design was applied to assess the impact of pH, stirring speed, and temperature on triglyceride adsorption rate. One gram(M1) of activated bamboo charcoal was placed into a beaker, followed by the addition of a aqueous solution (100 ml), pH set as 5,7 and 9 respectively, adjusted by HCI and KOH, containing five grams(m1) of triglycerides (purchased from Guangzhou Yongsheng Industry and Trade Co., Ltd., Guangzhou, China). Stirring adsorption was conducted for 10 min with a water bothing and magnetic stirrer (DF-101S, Shanghai Lichen-BX Instrument Technology Co., Ltd., Shanghai, China). The stirring speed was set as 600 rpm, 800 rpm, 1000 rpm and the temperature during stirring and adsorption was set as 25 °C, 35 °C, 45 °C respectively. A design plan of the orthogonal factors and levels is presented in Table 1. The solution after adsorption was centrifuged at a speed of 5000 rpm for 10 min with centrifuge (TG16-WS, Hunan Xiangyi Laboratory Instrument Development Co., Ltd., Changsha, China). the triglycerides above the aqueous phase were extracted by a syringe and subsequently used for further measurements. The triglyceride adsorption rate was calculated by Eq. (1).

**Table 1:** Orthogonal factors and level tables.

Factor/level	pH	T (°C)	Stirring speed (r/min)
1	5	25	600
2	7	35	800
3	9	45	1000

The triglyceride adsorption rate was calculated as per Eq. (1).

1 $$ y = (m_{1} - m_{2} ){\text{/M}}_{1} $$

m_1_: Initial weight of triglycerides before adsorption, g; m_2_: Weight of triglycerides after adsorption, g; M_1_: Consumption amount of activated bamboo charcoal, g; y: Triglyceride adsorption rate, (%).

### Structural analysis of activated bamboo charcoal

The structural analysis encompassed both the normal bamboo charcoal (procured from Hunan Xinsheng Bamboo Industry Co., Ltd., Yiyang City, China) and the activated bamboo charcoal prepared as detailed in Section "Preparation of steam-activated bamboo charcoal".

#### Brunauer–Emmett–Teller (BET) specific surface area measurement

A fully automated physical adsorption instrument (ASAP2460, Micromeritics, Georgia, USA) facilitated the BET specific surface area measurement and pore size analysis. Nitrogen adsorption was employed for analysis of all pore sizes, involving sample preparation at 700 °C and subsequent degassing at 300 °C.

#### Fourier transform infrared spectroscopy (FTIR) analysis

Testing was conducted using a Fourier transform infrared spectrometer (Nicolet iN10, Thermo Fisher Scientific Inc., Massachusetts, USA). Samples were compressed into potassium bromide pellets and subjected to FTIR scanning within the range of 400–4000 cm^−1^.

#### Scanning electron microscopy (SEM) analysis

Field Emission Scanning Electron Microscopy (Apreo 2 SEM, Thermo Fisher Scientific Inc., Massachusetts, USA) was performed. Samples were evenly affixed to conductive adhesive, followed by vacuum treatment for approximately 15 min. Surface morphology observations were undertaken at magnifications of 10^3^, 10^4^, and 10^5^, operating under conditions of 3 kV voltage.

#### X-ray diffraction analysis (XRD)

X-ray diffraction analysis employed an X-ray diffractometer (Ultima IV, Rigaku Corporation, Tokyo, Japan). Using a copper target, wide-angle diffraction scanning was performed on the sample within the range of 5–85° at a scanning rate of 5°/min.

### Molecular dynamics simulation

**Figure 1:**
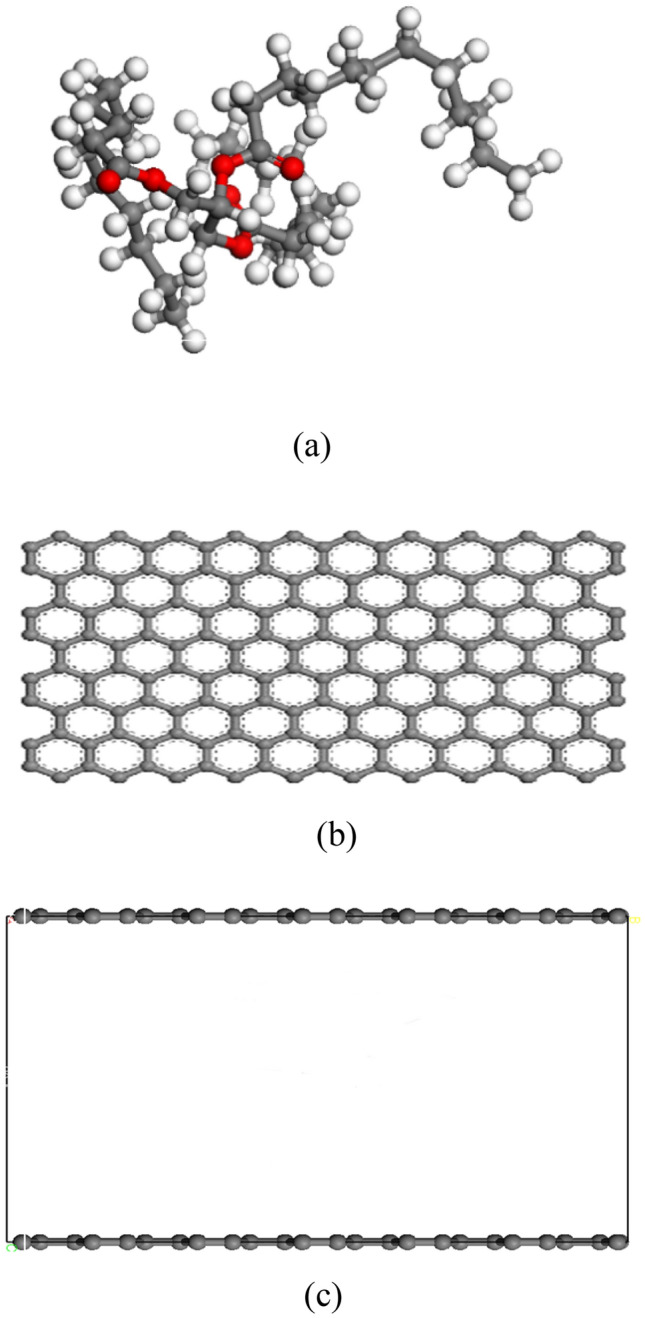
Models required for molecular dynamics simulation.

Activated bamboo charcoal, as described in this study, was used as the adsorbent, while triglyceride molecules served as adsorbates for simulation. The Materials Studio software (Accelrys Inc., New Jersey, USA), inclusive of the Forcite module and Sorption module, facilitated the simulation process. The triglyceride molecular structure, (CAS: 538–24-9, C_39_H_74_O6) as represented in Fig. 1a, was simulated. Bamboo charcoal, along with activated bamboo charcoal, was modeled using graphite sheets, following literature precedents, as shown in Fig. 1b ^19^. In accordance with literature recommendations, the pore structure of carbon materials was simulated through the utilization of two parallel graphite sheets, creating a slit model as demonstrated in Fig. 1c ^20^. The inter-layer distance and cell dimensions were determined based on the findings from Section "BET specific surface area measurement", leading to dimensions of 49.19 Å × 85.20 Å × 20 Å. The simulation system incorporated periodic boundary conditions. The Forcite module fac5ilitated the structural optimization of the constructed models to achieve the configuration with the lowest energy and maximum stability.

The Sorption module was employed to simulate adsorption with the Grand Canonical Monte Carlo (GCMC) technique on models of bamboo charcoal and activated bamboo charcoal. In the simulation, the COMPASS III force field was adopted, and the Adsorption Isotherm was selected as the Task. The simulation maintained a temperature of 308 K and a pressure range of 10 kPa to 1000 kPa. The total simulation steps encompassed 107 Monte Carlo steps, with 530 equilibration steps and 530 production steps, culminating in the generation of adsorption isotherms.

Molecular dynamics simulation relied on the Forcite module, utilizing the COMPASS Ш force field. Temperature control implemented the Nose algorithm, while pressure control followed the Berendsen method. Electrostatic interactions were evaluated through the Ewald summation method, and van der Waals interactions were calculated using the Atom-Based method. The time step was established at 1.0 fs. Initially, post-adsorption models underwent 50 cycles of annealing dynamics within the NVT ensemble. The configuration with the lowest energy was selected for NVT (isothermal-isobaric) dynamics simulation, extending for 1000 ps to enable the calculation of radial distribution functions.

## Results

### Triglyceride adsorption by activated bamboo charcoal

The results of triglyceride adsorption by activated bamboo charcoal are presented in Table 2, while the analysis of variance is shown in Table 3. Intuitive analysis of the orthogonal experimental results revealed that the highest adsorption rate of triglycerides by activated bamboo charcoal (27.0%) was achieved under conditions of a pH value of 5, temperature of 35°C, and a stirring speed of 800 r/min. The range (R) analysis indicated that the pH value had the most significant impact on the adsorption rate. The variance analysis table indicated the significant influence of pH and temperature on the adsorption rate. When tested under these conditions, the adsorption rate of triglycerides by bamboo charcoal was 4.4%, implying that activated bamboo charcoal exhibited 6.1 times higher triglyceride adsorption than ordinary bamboo charcoal.

**Table 2:** Orthogonal experimental results.

Serial number	pH	T (°C)	Stirring speed (r/min)	Blank	Adsorption/%
1	5	25	600	1	23.8
2	5	35	800	2	27.0
3	5	45	1000	3	24.2
4	7	25	800	3	25.8
5	7	35	1000	1	25.2
6	7	45	600	2	22.2
7	9	25	1000	2	18.2
8	9	35	600	3	17.4
9	9	45	800	1	19.2
K1	25.0	22.6	21.1	22.7	
K2	24.4	23.2	24.0	22.5	
K3	18.3	21.9	22.5	22.5	
R	6.7	1.3	2.9	0.3

**Table 3:** Analysis of variance table.

Factor	F	Degree of freedom	F ratio	Significance
pH	83.32	2	586.73	*
Temperature	2.68	2	18.84	
Rotate speed	12.33	2	86.82	*
Error	0.14	2		

*P < 0.05 and F0.05 (2, 2) = 19.00.

The use of activated biomass charcoal for oil adsorption has been reported in some fields. Del Angel et al. achieved 94% oil removal from domestic wastewater using sugarcane bagasse biochar activated with H_3_PO_4_^21^. The activation of crab shell biochar with KOH increased the specific surface area of biochar by 93-fold, enhancing its oil adsorption capacity in diesel-contaminated water^21^. Activated date palm biochar increased its specific surface area by 741.5 m2/g, removing 95.2% organic compounds^22^ from oil-containing wastewater. These findings underscore the efficiency of activated biochar for oil adsorption. In comparison, the steam activation method employed in this study offers a simple production process for biomass-activated carbon and avoids the introduction of harmful impurities^17^, making it particularly suitable for the cosmetics industry.

### BET specific surface area measurement

The measured data are shown in Table 4. The BET-calculated surface area of activated bamboo charcoal was 560.0 m2/g, whereas that of ordinary bamboo charcoal was 4.9 m2/g. Activated bamboo charcoal exhibited a specific surface area 114.2 times greater than that of ordinary bamboo charcoal. The average pore size of activated bamboo charcoal is 1.6 nm, and the average pore size of bamboo charcoal is 7.2 nm.

**Table 4:** BET measurement data.

Sample	Surface area (m^2^/g)	Total pore volume (m^3^/g)	Type of adsorption isotherm	Average pore size/nm	Micropore volume (mm^3^/g)
Activated bamboo charcoal	560.0	230.0	I	1.6	200.0
Bamboo charcoal	4.9	8.8	III	7.2	1.8

**Figure 2:**
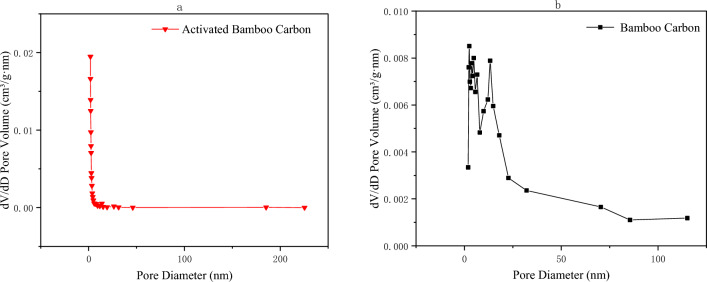
Aperture distribution curves analyzed by BJH method by: (**a**) activated bamboo charcoal and (**b**) ordinary bamboo charcoal.

Figure 2 shows the pore size distribution curve analyzed by the Barrett-Joyner-Halenda (BJH) single-point method. According to analysis, the total pore volume of activated bamboo charcoal was 230.0 mm3/g, with a micropore volume of 200.0 mm3/g, accounting for 86.9% of the total pore volume. In contrast, ordinary bamboo charcoal had a total pore volume of 8.8 mm3/g, with a micropore volume of 1.8 mm3/g, constituting 20.4% of the total pore volume. These findings indicate a significant increase in the quantity of micropores following steam activation, with the total pore volume of activated bamboo charcoal being 26.14 times that of ordinary bamboo charcoal.

**Figure 3:**
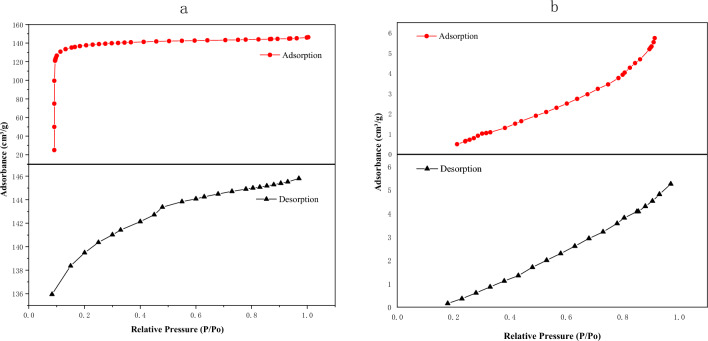
BET adsorption desorption curves by: (**a**) activated bamboo charcoal and (**b**) ordinary bamboo charcoal.

Figure 3a represents the nitrogen adsorption–desorption isotherm of activated bamboo charcoal. The adsorption isotherm belongs to Type I according to the Brunauer-Deming-Deming-Teller classification. In the low P/P0 range, the curve exhibits an upward convex shape, while in the high P/P0 range, the isotherm remains relatively flat. These observations suggest the presence of numerous pore structures in the tested activated bamboo charcoal sample, with pore sizes predominantly distributed in the micropore area. Figure 3b represents the nitrogen adsorption–desorption isotherm of ordinary bamboo charcoal. The adsorption isotherm falls into Type Ш as per the Brunauer-Deming-Deming-Teller^23^ classification. Notably, there is no distinct saturated adsorption plateau, indicating that the surface pore structure of unmodified bamboo charcoal is highly irregular.

### Fourier transform infrared spectroscopy

**Figure 4:**
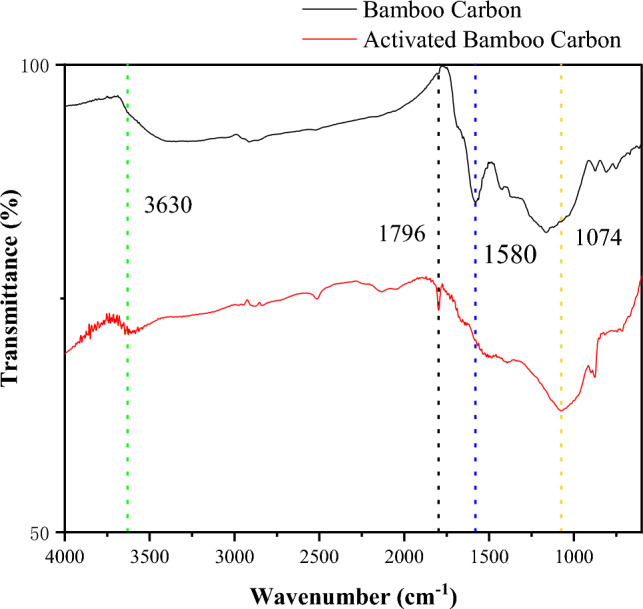
Infrared spectrogram.

The FTIR analysis results are shown in Fig. 4. It can be seen from the figure that the peak shapes of the infrared spectra of ordinary bamboo charcoal and activated bamboo charcoal are roughly the same. These results indicate that steam activation is a physical process that does not alter the surface's chemical properties^24^. The spectra reveal that the stretching vibration at wavenumber 1580 cm^−1^ in ordinary bamboo charcoal is attributed to the stretching vibration of the nearly aromatic ring^25^. For activated bamboo charcoal, this vibration is significantly reduced, indicating a substantial removal of hydrogen elements. The broad absorption peak at wavenumber 3630 cm^−1^ suggests the presence of hydroxyl group (O–H) stretching vibrations, and the absorption peak at wavenumber 1074 cm^−1^ suggests the existence of C–O–C and C–OH stretching, indicating the residual oxygen-containing functional groups after cellulose pyrolysis^26^. In the literature, more oxygen-containing groups are inducted to the surface of bamboo charcoal by boric acid (H_3_BO_3_) activated mothed^27^, phosphorus element are inducted to the carbon material made from cotton straw by phosphoric acid (H_3_PO_4_) activated^28^, which may cause safety hazards to the skin, while the bamboo charcoal activated by steam does not induct more impurity elements.

### Scanning electron microscopy analysis

**Figure 5:**
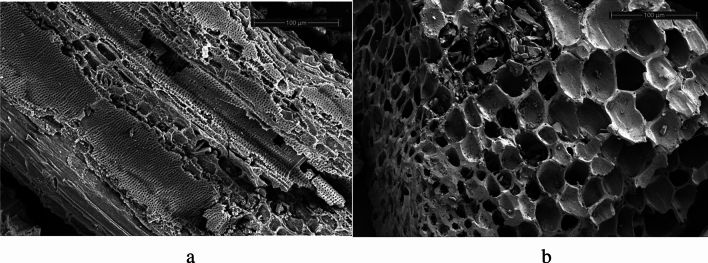
SEM Images of the samples at 1000 magnification: (**a**) ordinary bamboo charcoal (**b**) activated bamboo charcoal.

Figure 5 presents the SEM images of activated bamboo charcoal at different magnifications figure. The image reveals inherent pores and a relatively rough surface structure in activated bamboo charcoal. Debris is observed in the pores, and a substantial number of microporous structures are distributed on the pore walls, which are formed through steam activation. The increased number of pores after steam activation enhance the performance of modified bamboo charcoal in the adsorption process. According to the SEM images described in the literature, steam activation will make the surface of eucalyptus wood chips smooth, wrinkled and open^29^. Many pores of different sizes are produced on steam activated desilicated rice husk^30^. These show that steam activation can significantly increase the number of pores.

### X-ray diffraction

**Figure 6:**
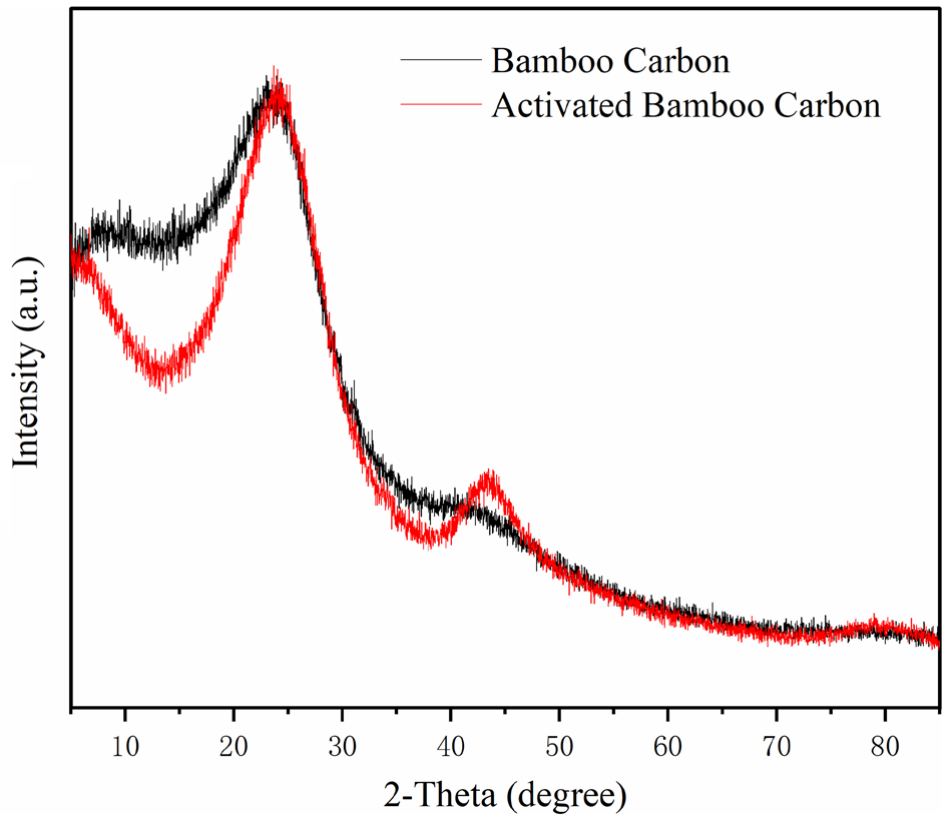
XRD patterns of sample using: (**a**) ordinary bamboo charcoal and (**b**) activated bamboo charcoal.

XRD patterns of ordinary bamboo charcoal and activated bamboo charcoal are shown in Fig. 6. The diffraction peaks at 2θ = 23° for both materials indicate the (002) crystalline planes of graphite microcrystalline structure, present before and after activation. According to the literature, the carbon structure of activated carbon products is mainly amorphous, with some crystalline material present^31^. the diffraction peak at 2θ = 42° for activated bamboo charcoal exhibits a significantly higher height and width compared to ordinary bamboo charcoal, representing the (100) crystalline planes of graphite-like microcrystalline structure, and the diffraction peak at 2θ = 23° is improved. This suggests an increased degree of graphitization and smaller microcrystalline size in activated bamboo charcoal, leading to the formation of abundant microporous structures. Compared with the XRD pattern of activated carbon prepared from coconut shell and coal^32^, activated bamboo charcoal has only two obvious diffraction peaks, indicating that activated bamboo charcoal has no obvious impurities.

### Intermolecular interaction energy of triglyceride adsorption by activated bamboo charcoal

The strength of intermolecular interactions can be compared through interaction energy. According to thermodynamic theory, the calculation equation^33^ for interaction energy (ΔE) is as follows:

2 $$ \Delta E = E_{t} - ({\text{E}}_{{{\text{tm}}}} + {\text{E}}_{{{\text{cl}}}} ) $$

where E_t_ represents the total energy of interaction between triglyceride molecules and the charcoal layer, E_tm_ is the energy of triglyceride molecules, and E_cl_ is the energy of the charcoal layer. A positive ΔE indicates a repulsive force between the two substances, while a negative ΔE represents an attractive force.

The calculated interaction energy between the activated bamboo charcoal layer and the triglyceride molecules is − 145.12 kcal/mol, whereas the interaction energy between the bamboo charcoal layer and the triglyceride molecules is − 132.73 kcal/mol. This indicates that both ordinary bamboo charcoal and activated bamboo charcoal exhibit attractive forces towards triglyceride molecules. Furthermore, the interaction energy of the activated bamboo charcoal layer with triglyceride molecules is 12.39 kcal/mol higher than that of the ordinary bamboo charcoal layer, suggesting that ordinary bamboo charcoal after activation has a stronger adsorption capacity for triglyceride molecules, consistent with the findings in Section "Preparation of steam-activated bamboo charcoal". Furthermore, it has been noted in the literature that An et al. conducted molecular simulations to investigate the adsorption of various organic compounds on activated carbons with different pore sizes. They found that a reduction in pore size by 4 nm corresponds to an increase in interaction energy of approximately 4 kcal/mol^20^. This suggests that a decrease in pore size can enhance adsorption capability.

**Figure 7:**
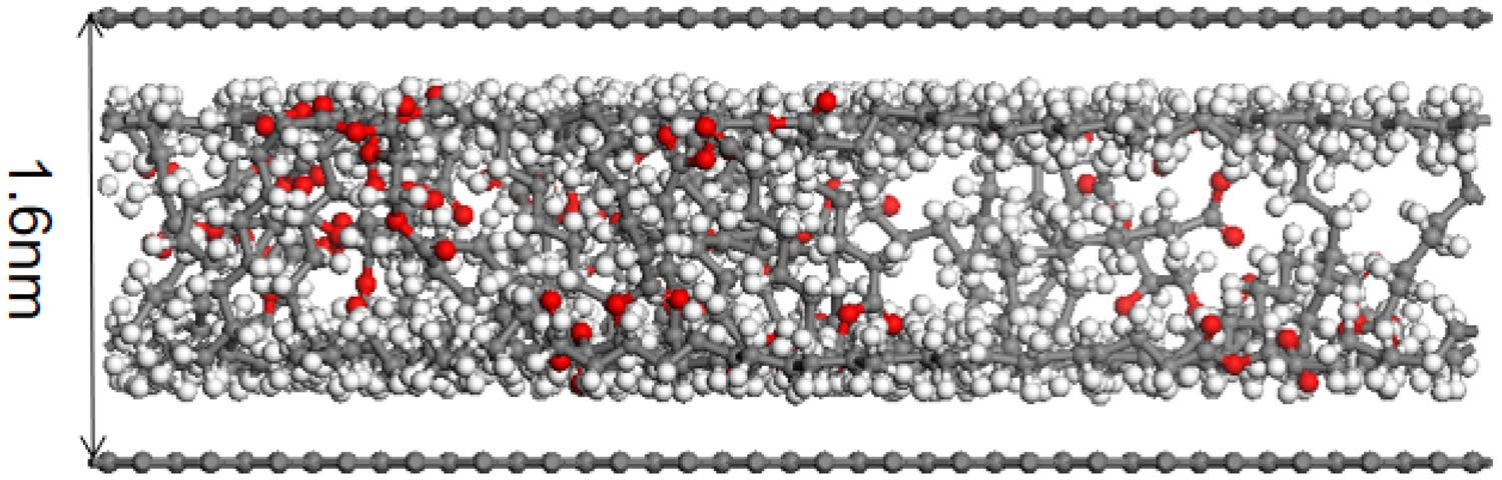
Simulated adsorption of triglyceride molecules by the 1.6 nm activated bamboo charcoal layer.

**Figure 8:**
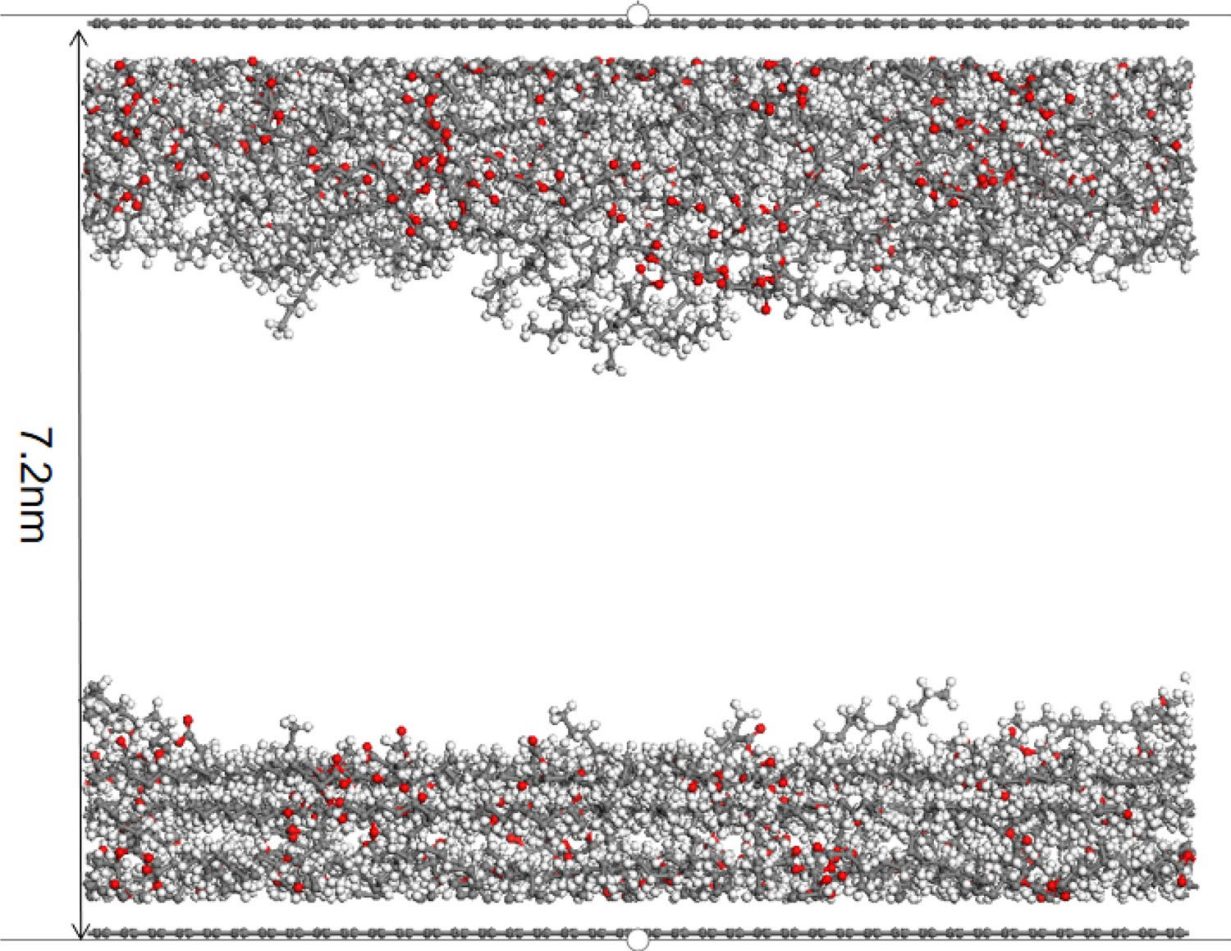
Simulated adsorption of triglyceride molecules by the 7.2 nm bamboo charcoal layer.

The simulated adsorption of triglyceride molecules by the activated bamboo charcoal layer (pore size 1.6 nm) and the ordinary bamboo charcoal layer (pore size 7.2 nm) is shown in the Figs. 7 and 8. After reaching adsorption equilibrium, triglyceride molecules are observed to migrate closer to the pore walls and become adsorbed. Due to its smaller pore size, activated bamboo charcoal exhibits stronger aggregation of triglyceride molecules within the pores, resulting in more efficient adsorption compared to ordinary bamboo charcoal. It is because that the surface attraction of the pore walls adjacent overlaps and enhances in the pore. When the pore size decreases, the adsorption capacity to triglycerides of the pore increases. Due to the larger pore size of ordinary bamboo charcoal, the diffusion of triglyceride molecules in the pores of ordinary bamboo charcoal is greater than that of activated bamboo charcoal. Triglyceride molecules exhibits weaker aggregation of further away from the pore walls of ordinary bamboo charcoal, making them less prone to adsorption. Consistent with the results presented in Table 4, activated bamboo charcoal demonstrates significantly higher triglyceride adsorption rates than ordinary bamboo charcoal, affirming the agreement between molecular dynamics simulation and practical outcomes. According to Artur P. Terzyk’s simulation results of study, the microporosity rises for a larger number of phenol molecules is adsorbed in smaller micropores on the microporous carbons. In one porous carbon models, total adsorption decreases for the decrease in the pore volumes^34^. This is consistent with the results of this paper.

### Adsorption isotherm of triglyceride adsorption by activated bamboo charcoal

**Figure 9:**
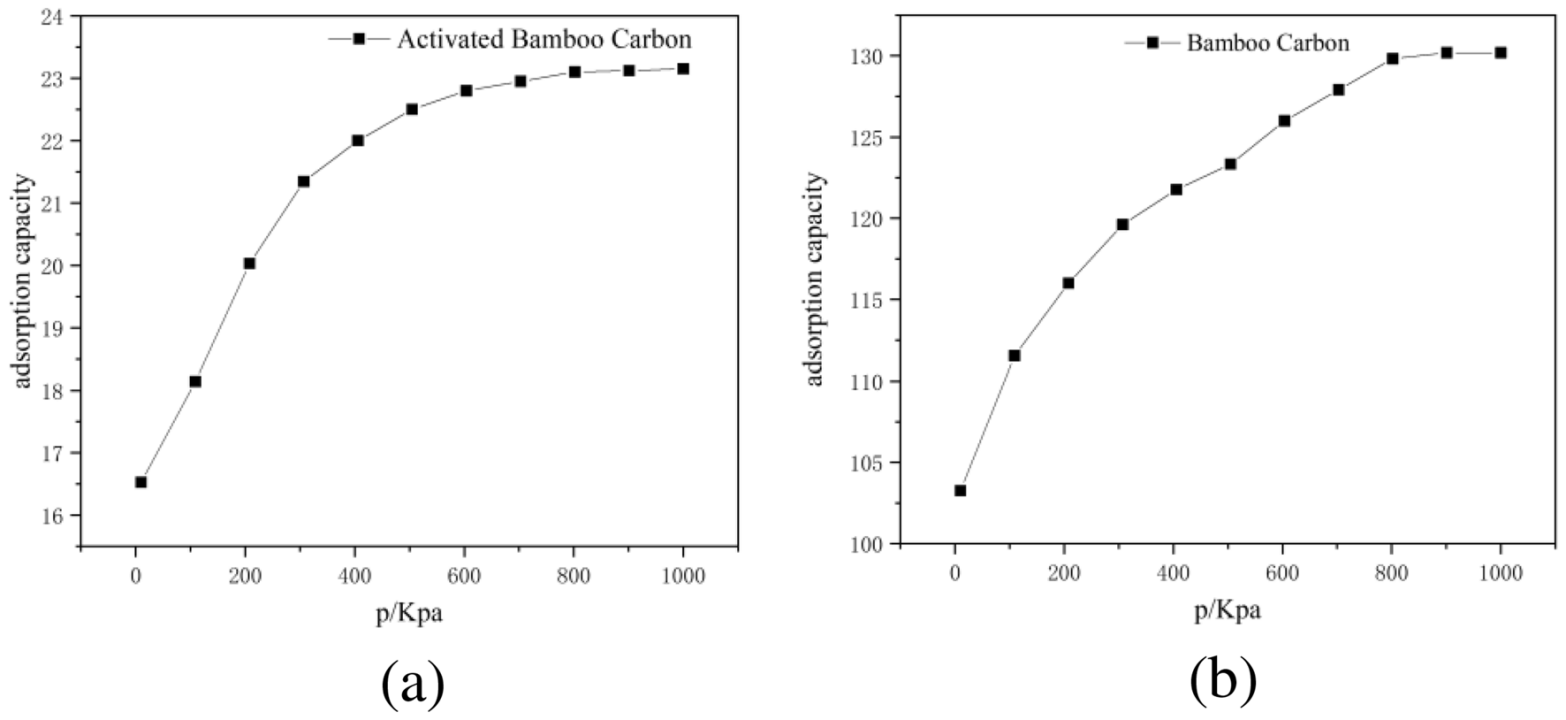
Adsorption isotherm for the simulated unit carbon molecular layer adsorbing triglyceride molecules using: (**a**) activated bamboo charcoal and (**b**) ordinary bamboo charcoal.

The adsorption isotherm refers to the curve depicting the variation of adsorption quantity and adsorption pressure when an adsorbate is adsorbed at the interface of two phases at a specific temperature. The adsorption isotherm for the simulated unit carbon molecular layer adsorbing triglyceride molecules is shown in Fig. 9.

According to Fig. 9, at adsorption equilibrium, each simulated unit of activated bamboo charcoal layer can adsorb a maximum of 23 triglyceride molecules, while each simulated unit of ordinary bamboo charcoal layer can adsorb up to 130 triglyceride molecules. Although the calculated triglycerides adsorption by activated bamboo charcoal is less than ordinary bamboo charcoal when considering a single unit of charcoal layer, the calculations based on specific surface area and pore size reveal that activated bamboo charcoal, with 2.78 × 10^20^ pores per gram, can adsorb approximately 1.77 × 10^21^ triglyceride molecules per gram. In contrast, ordinary bamboo charcoal, with 1.20 × 10^17^ pores per gram, can adsorb only about 1.56 × 10^19^ triglyceride molecules per gram. This suggests that although activated bamboo charcoal has smaller pores and lower individual pore adsorption capacity, its increased pore quantity enhances its overall adsorption capacity for triglyceride molecules compared to ordinary bamboo charcoal. Therefore, activated bamboo charcoal proves to be a more effective cosmetic ingredient than ordinary bamboo charcoal for sebum adsorption.

### Radial distribution function of triglyceride adsorption by activated bamboo charcoal

The radial distribution function (RDF) describes the probability of identifying another particle at a certain distance from a reference particle. In this study, the distance between charcoal layer molecules and triglyceride molecules can be determined. The calculation equation is as follows^35^:

3 $$ g_{(A - B)} (r) = \frac{1}{{4\pi \rho Br^{2} }}*\frac{{dN_{A - B} }}{dr} $$

where ρB represents the number density of Particles B in the model; N_A-B_ stands for the number of Particles B within a radius range r-(r + dr) with Particles A at the center; g_A-B_(r), denoted as RDF, represents the relative probability of triglyceride molecules appearing at different distances from the carbon layer molecules, with r representing the distance between triglyceride molecules and carbon layer molecules.

**Figure 10:**
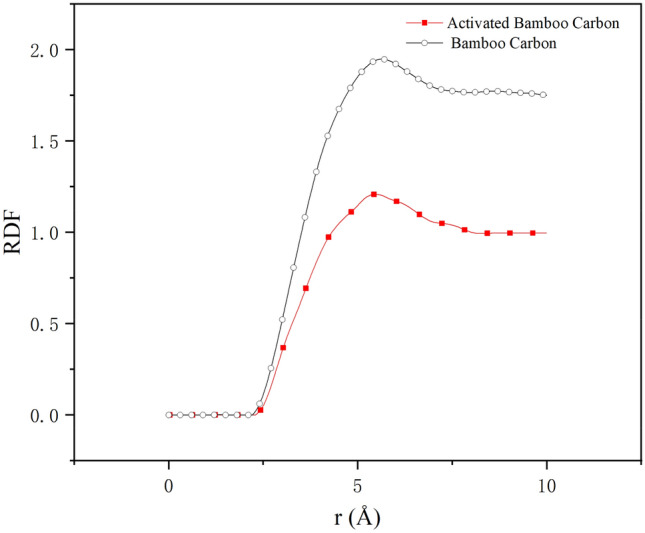
RDF curves between the carbon molecular layer and triglyceride molecules.

The RDF curves between the carbon molecular layer and triglyceride molecules are shown in Fig. 10. The RDF curves for activated bamboo charcoal and ordinary bamboo charcoal both exhibit their maximum peaks in the range of 5 to 6 Å, i.e., between 0.5 and 0.6 nm. The peak value for ordinary bamboo charcoal is 1.95, while for activated bamboo charcoal, it is 1.21. Moreover, the overall curve for ordinary bamboo charcoal is higher than that for activated bamboo charcoal. This indicates that the carbon molecular layer of bamboo charcoal adsorbs a greater quantity of triglyceride molecules compared to the carbon layer of activated bamboo charcoal, consistent with the results from Section "Adsorption isotherm of triglyceride adsorption by activated bamboo charcoal".

## Conclusions

In this study, bamboo charcoal was activated by steam, which can physically adsorb triglycerides. steam-activated bamboo charcoal exhibited an adsorption rate of up to 27.0% for triglycerides, which was 6.1 times higher than that of ordinary bamboo charcoal. After activation, the specific surface area of activated bamboo charcoal was 560.0 m^2^/g, which was 114 times than that of ordinary bamboo charcoal. Average pore size reduced from 7.2 nm to 1.6 nm. According to the specific surface area and pore diameter, the number of pores increased by 2317 times. Molecular dynamics simulations of the adsorption process revealed that the interaction energy between the carbon molecular layer and triglyceride molecules increased from − 132.73 kcal/mol to − 145.12 kcal/mol after activation, According to the number of triglyceride molecules adsorbed per unit activated carbon molecular layer obtained from the adsorption isotherm, the number of triglyceride molecules adsorbed per gram of activated bamboo charcoal was calculated to be 1.77 × 10^21^, while ordinary bamboo charcoal was only 1.56 × 10^19^. It indicated that the strong triglyceride adsorption capacity of steam-activated bamboo charcoal is achieved through increased intermolecular interaction energy and a substantial increase in the number of pores. The research results of this article provide a biomass material that can reduce the amount of sebum based on physical adsorption to the cosmetics industry. In the future, we will further evaluate the safety through human experimentation.

## Data Availability

The datasets used and/or analysed during the current study available from the corresponding author on reasonable request.
